# Supplementary material for: Laser activation of single group-IV colour centres in diamond
Source: Nat Commun. 2025 Jun 2;16:5124. doi: 10.1038/s41467-025-60373-5 (PMC12130254; doi:10.1038/s41467-025-60373-5)
Supplement: Supplementary file 1 — Supplementary Information [file 41467_2025_60373_MOESM1_ESM.pdf]

Laser Activation of Single Group-IV Colour Centres in Diamond

Xingrui Cheng,[1,2] Andreas Thurn,[3,2] Guangzhao Chen,[1,*] Gareth S. Jones,[1] James E. Bennett,[1] Maddison Coke,[4] Mason Adshead,[4,5] Cathryn P. Michaels,[3] Osman Balci,[6] Andrea C. Ferrari,[6] Mete Atatüre,[3] Richard J. Curry,[4,5] Jason M. Smith,[1,†] Patrick S. Salter,[2,‡] and Dorian A. Gangloff[3,2,§]

[1]*Department of Materials, University of Oxford, Parks Road, Oxford OX1 3PH, UK*
[2]*Department of Engineering Science, University of Oxford, Parks Road, Oxford OX1 3PJ, UK*
[3]*Cavendish Laboratory, University of Cambridge, J. J. Thomson Avenue, Cambridge CB3 0HE, UK*
[4]*Photon Science Institute, Faculty of Science and Engineering, University of Manchester, Manchester M13 9PL, UK*
[5]*Department of Electrical and Electronic Engineering, Faculty of Science and Engineering, University of Manchester, Manchester M13 9PL, UK*
[6]*Department of Engineering, University of Cambridge, Trumpington Street, Cambridge CB2 1PZ, UK*

Supplementary Information

Supplementary Note 1: Heat diffusion simulation

For the laser annealing process it is useful to have an estimate for the timescales on which heat diffusion happens in diamond. One limitation of such a simulation is that, for diamond, the multi-photon absorption coefficients for femtosecond laser pulses at 520 nm are not well known experimentally. This means that it is hard to properly estimate the energy that is delivered into the focal volume and therefore available to heat the diamond lattice.

However, despite the above limitation, it is still instructive to use a thermal diffusion equation to get an intuition for the rate at which heat diffusion happens in diamond given certain initial conditions. For a heat profile $T(\mathbf{r}, t)$ given as a function of position $\mathbf{r}$ and time t, the diffusion equation is given by

$$\frac{\partial T(\mathbf{r}, t)}{\partial t} = \alpha \nabla^2 T(\mathbf{r}, t), \tag{1}$$

where $\alpha = k/(\rho c_\mathrm{p})$ is the thermal diffusivity, k the thermal conductivity, ρ the density and c_p the specific heat. For the initial temperature distribution, we used a three dimensional gaussian

$$T(\mathbf{r}, 0) = T_\mathrm{b} + (T_\mathrm{h} - T_\mathrm{b}) \exp\left[-\left(\frac{x^2}{2\sigma_\mathrm{x}^2} + \frac{y^2}{2\sigma_\mathrm{y}^2} + \frac{z^2}{2\sigma_\mathrm{z}^2}\right)\right], \tag{2}$$

with standard deviations σ_x, σ_y, σ_z given by the focusing conditions of the laser beam (see Methods). For the initial peak temperature, we chose a value of $T_\mathrm{h} = 1050\,°\mathrm{C}$ as an upper limit, motivated by the fact that diamond starts to graphitise at such a temperature under steady-state conditions [1]. The absence of graphitisation in our experiments, despite tests with >12 h of exposure, suggests that our actual peak temperatures remain well below this estimate. The ambient background temperature was set to $T_\mathrm{b} = 25\,°\mathrm{C}$. The time evolution of the initial temperature distribution can be calculated by convolution with the corresponding Green's function.

In the relevant temperature range, the thermal diffusivity of diamond decreases with increasing lattice temperature. For analytic solutions, we therefore consider two cases, room temperature, and high temperature ($\sim 930°$C, material parameters for even higher temperatures were not available). Using room temperature parameters yields a diffusivity of $\alpha_\mathrm{rt} = 1.22 \cdot 10^{-3}\,\mathrm{m}^2/\mathrm{s}$ [2, 3] and overestimates the cooling rate. Using high temperature parameters yields a diffusivity of $\alpha_\mathrm{hot} = 0.06 \cdot 10^{-3}\,\mathrm{m}^2/\mathrm{s}$ [2, 3] and underestimates the cooling rate. The real temperature dependence is therefore bounded by those two cases, which provides a good enough estimate for our purposes here.

The results of this calculation are summarised in supplementary Fig. 1 below. Supplementary Fig. 1a shows a top view of the initial lateral temperature distribution. The optical axis is along the z-axis. Supplementary Fig. 1b presents the temperature profiles along the x-axis at successive points in time, showing the diffusion of the temperature

* Current address: Accelerator Technology and Applied Physics Division, Lawrence Berkeley National Laboratory, Berkeley, California 94720, USA
† Corresponding author: jason.smith@materials.ox.ac.uk
‡ Corresponding author: patrick.salter@eng.ox.ac.uk
§ Corresponding author: dag50@cam.ac.uk

distribution. Supplementary Fig. 1c shows the maximum temperature as a function of time for both cases of thermal diffusivity we are considering. From the results it is evident that the heat dissipates within $\sim 10\,\mathrm{ns}$ in either case, two orders of magnitude faster than the time between two consecutive annealing pulses (1 µs). This rapid cooling shows that no cumulative heating is expected in our experiments. Supplementary Fig. 1d shows the spread of the temperature distribution within the first nanosecond. It can be seen that the temperature distribution spreads out at most to a few micrometers in diameter before almost cooling down to ambient conditions.

This is consistent with our observation, see supplementary Fig. 6, where extended laser annealing affected a region of several micrometers in diameter. Further experimental research has been conducted into heat dissipation in diamond following pulsed femtosecond excitation at near infrared wavelengths [4]. The authors observed heat dissipation over a similar sized region within a few nanoseconds [4], consistent with our results.

It is further interesting to note that the energy contained in the temperature distribution shown in supplementary Fig. 1 is 0.14 nJ, which for a 1 nJ input pulse - as we used in our experiments - would correspond to 14 % absorption. In reality, the absorption is likely much lower. While the three-photon absorption coefficient β_3 at 520 nm has not been experimentally determined, measurements indicate that $\beta_{3,520\mathrm{nm}} << \beta_{3,400\mathrm{nm}} = 2.3 \cdot 10^{-22}\,\mathrm{cm^3W^{-2}}$ [5]. Using $\beta_{3,400\mathrm{nm}}$ as an upper bound, we can use [5]

$$\frac{\Delta I}{\Delta z} \approx -\beta_{3,400\mathrm{nm}} I_0^3 \tag{3}$$

to estimate the fraction of absorbed pulse energy as

$$\frac{\Delta E}{E_0} = \frac{\Delta I}{I_0} \approx -\beta_{3,400\mathrm{nm}} I_0^2 \Delta z = 16\,\%. \tag{4}$$

Here, the effective interaction length $\Delta z = 790\,\mathrm{nm}$ is taken as the confocal parameter, $E_0 = 1\,\mathrm{nJ}$ is the initial pulse energy, $I_0 = 3 \cdot 10^{12}\,\mathrm{Wcm^{-2}}$ is the initial peak intensity, and ΔE, ΔI_0 are the respective changes in pulse energy and peak intensity. The peak intensity is given by $I_0 \approx F/\tau$, where $F = 1.2\,\mathrm{J\,cm^{-2}}$ is the fluence and $\tau = 400\,\mathrm{fs}$ the pulse duration. For our experimental conditions, the expected fraction of absorbed pulse energy is therefore $<< 14 - 16\,\%$, which means that it is to be expected that the maximum temperatures reached in our experiments are significantly lower than $T_\mathrm{h} = 1050\,°\mathrm{C}$.

Supplementary Note 2: Activated SnV^- positioning accuracy

A PL map of the diamond sample was analysed to quantify the positioning accuracy of the activated Sn-related centre array. Array with an implantation dose of 500 ions per site was selected for this study because the sites can be nearly fully activated by the femtosecond laser without the activated centres becoming too bright. The analysis focused on a central 5 µm x 5 µm area of the activated array. The colourbar limits were chosen such as to only show the implantation sites and not the background. Gaussian fits were applied to each individual emitter within the region of interest (ROI), with the centre of each fitted Gaussian being considered as the emitter's position.

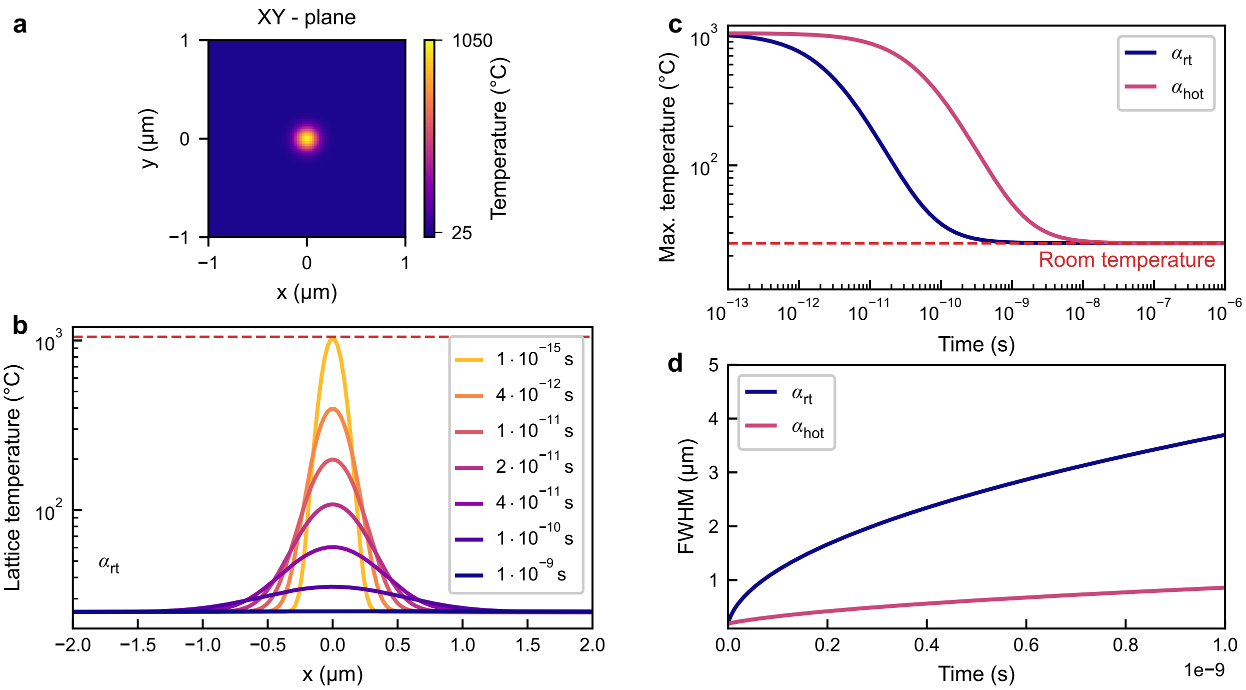

Supplementary Fig. 1: **Simulation of spatiotemporal thermal dynamics in ultrapure diamond.** **a**, Cross-sectional temperature distribution in the XY plane immediately after excitation. The initial peak temperature is set to 1050 °C. At such a temperature, diamond graphitises within minutes under steady-state conditions. As no graphitisation is observed over extended periods, the peak temperatures reached in our study are likely significantly lower. **b**, X-axis temperature profiles at successive timepoints assuming room-temperature (α_{rt}) diffusivity, demonstrating nanosecond-scale thermal equilibration. **c**, Maximum temperature as a function of time for room-temperature (α_{rt}) and high-temperature (α_{hot}) diffusivity parameters. **d**, Time evolution of the full width at half maximum (FWHM) for both cases.

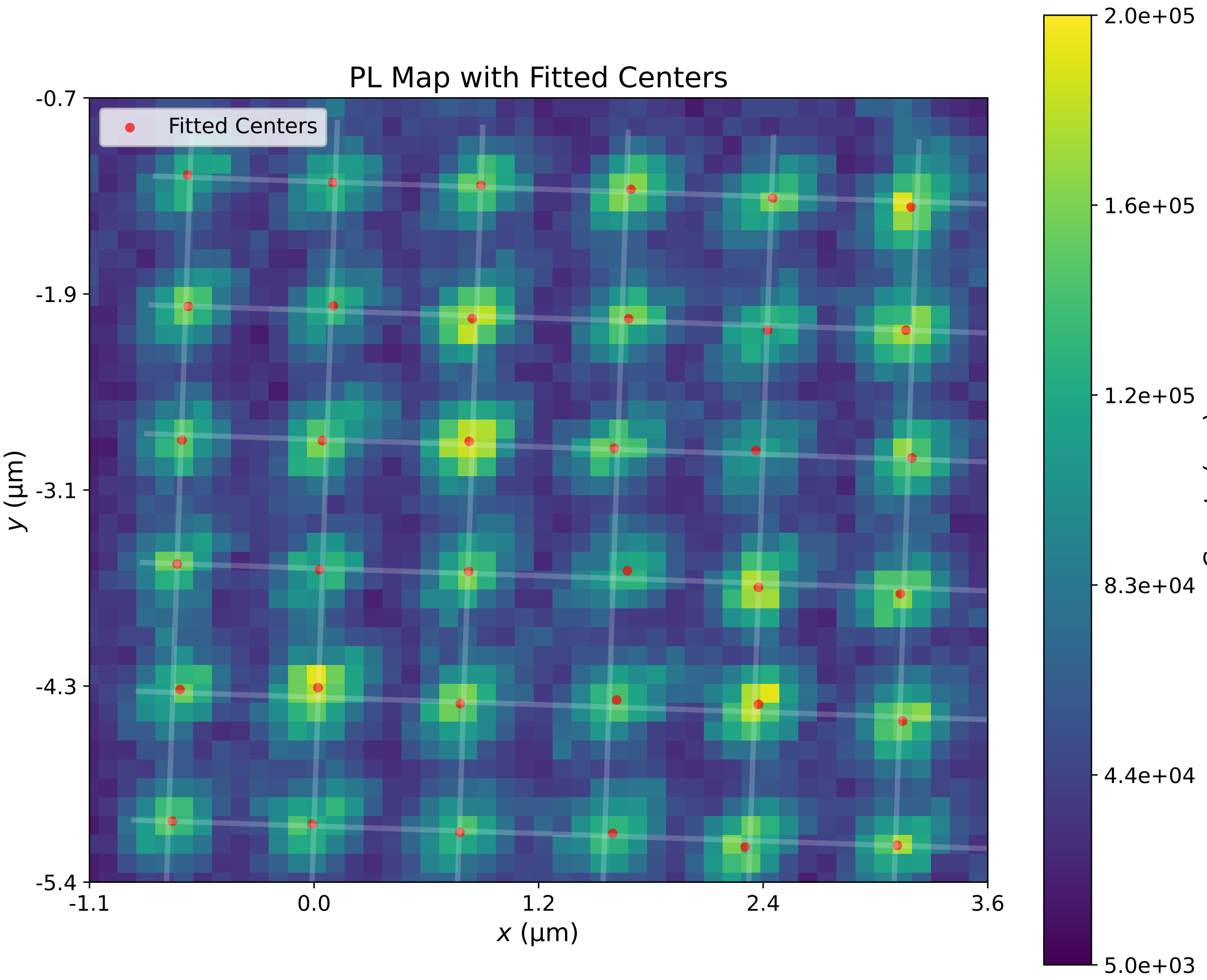

Supplementary Fig. 2: **Photoluminescence (PL) image of a post-laser activation Sn-implanted array**. 2D PL map of an array implanted with approximately 500 Sn ions per site, following laser activation. The white grid lines correspond to the theoretically fitted positions according to the implantation mask, and the red dots correspond to centre of the fitted Gaussian of individual emitter.

$$f(x,y) = \text{offset} + A \times \exp\left(-\left(\frac{(x-x_0)^2}{2\sigma_{\rm x}^2}\right) - \left(\frac{(y-y_0)^2}{2\sigma_{\rm y}^2}\right)\right), \tag{5}$$

where *offset* is the constant background level, A is the peak height of the Gaussian, x_0 and y_0 are the coordinates of the centre of the Gaussian, and $\sigma_{\rm x}$ and $\sigma_{\rm y}$ are the standard deviations in the x and y directions, respectively.

An ideal grid with a known uniform spacing of 0.78 µm, derived from the implantation mask, was fitted to the observed Sn implanted positions by optimising the x-offset (dx), y-offset (dy), and rotation angle (θ). The optimisation minimised the total discrepancy:

$$D = \sum_{\rm i} w_{\rm i} \min_{\rm j} \sqrt{(x_{0,\rm i} - x'_{\rm j})^2 + (y_{0,\rm i} - y'_{\rm j})^2} \tag{6}$$

where $(x_{0,\rm i}, y_{0,\rm i})$ are the fitted Gaussian centres of the emitters, and $(x'_{\rm j}, y'_{\rm j})$ are the nearest points on the ideal grid. Additionally, the radial discrepancy ($D_{\rm r}$) was calculated for each centre based on the offsets in (x) and (y), where

$$D_{\mathrm{r}} = \min_{\mathrm{j}} \sqrt{(x_0 - x'_{\mathrm{j}})^2 + (y_0 - y'_{\mathrm{j}})^2}, \tag{7}$$

This radial discrepancy (D_{r}) represents the distance between each observed centre and the ideal grid, providing a measure of how closely the observed centres align with the expected positions after accounting for the optimised offsets.

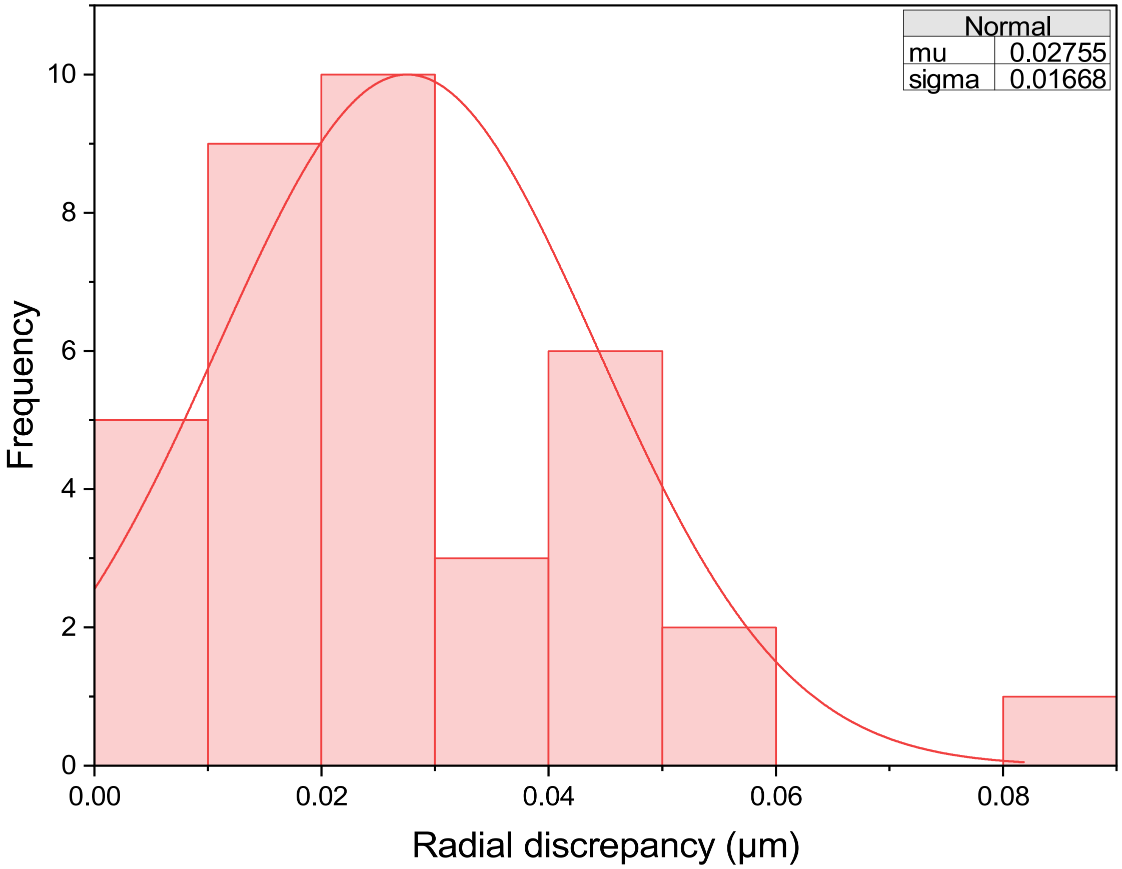

Supplementary Fig. 3: **Histogram of radial discrepancies**. The histogram shows the distribution of radical discrepancies, with normal distribution fits overlaid. The fitted mean and standard deviation for the radial discrepancy are 27.6 nm and 16.9 nm, respectively.

Analysis of the optimised discrepancies revealed an average radial discrepancy of 27.6 nm. The sub-30 nm average discrepancy demonstrates the high precision of our combined implantation and activation process, closely aligning with the expected implantation positioning accuracy. Notably, the laser activation process does not alter the position of the implanted Sn atoms. According to the P−NAME implantation system parameters used in this study, the positioning accuracy for the ion implantation is 100 nm, The observed accuracy may exceed expectations due to the high dosage region used, where each bright emitter represents an ensemble of defects. In this context, Gaussian fitting averages the distribution of ions, which will enhance the precision estimate. To achieve a more representative measure, we applied the same method to a 10 ions/site array, where laser-activated emitters (SnV^- or Type II Sn) typically appeared as singlets, doublets, or triplets as discussed in the previous section, providing a more accurate assessment of individual positioning accuracy. Analysis for the 10 ions/site array yields an average radial discrepancy of 150 nm and standard deviation of 90 nm, which is still comparable to the P−NAME implantation system's set resolution in this study.

Supplementary Note 3: Effect of pulse energy and annealing time

To gain deeper insight into the laser annealing process, we studied the effects of annealing laser pulse energy and annealing time on the activation of implanted emitters. Supplementary Fig. 4 presents a series of PL maps of the same site within a region implanted with 1000 ions per site. The site was annealed for 30 minutes with a pulse energy of 1.05 nJ, with the laser focused on the red circled points, which remained unchanged throughout the process. The annealing was divided into six distinct intervals, with PL images captured at each interval under identical experimental conditions. We note that in supplementary Fig. 4 the colour scale is set to the same upper limit in all plots to better visualise the radial spread of the activation, and thereby causing the PL intensity at the centre of the laser focus to appear saturated.

To quantify the spread and intensity of the activated area, a Gaussian function was fitted to the PL maps, treating PL intensity (here we are collecting emissions from both Sn-related defects and GR1s, and the figures displayed a min and max of brightness for better visibility, no capping was applied for any analyses) as the Gaussian height and analysing the spread across the x and y axes. The full width at half maximum (FWHM) and Gaussian peak height were extracted and plotted against annealing time, as shown in supplementary Fig. 6.

The FWHM along the x and y axes was determined by:

$$\mathrm{FWHM}_{\mathrm{x/y}} = 2\sqrt{2\ln 2} \times \sigma_{\mathrm{x/y}}. \tag{8}$$

An average FWHM was calculated, and the peak height was taken as I.

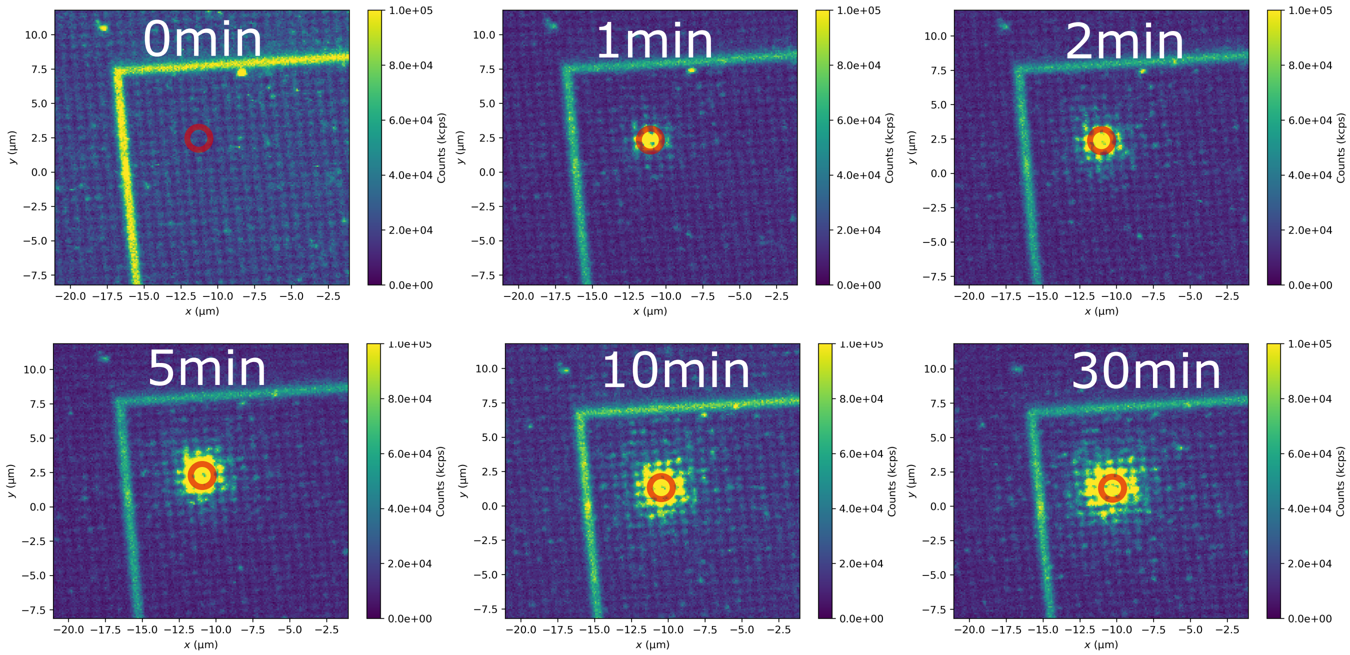

Supplementary Fig. 4: **Study of the effects of laser annealing time**. Sets of 2D photoluminescence (PL) images are shown for a region implanted with 1000 ions per site, taken at the same locations after varying annealing times (annotated in white within each PL map). The laser was focused on the red circled points in the PL maps, with a slight defocus of 1 μm from the surface.

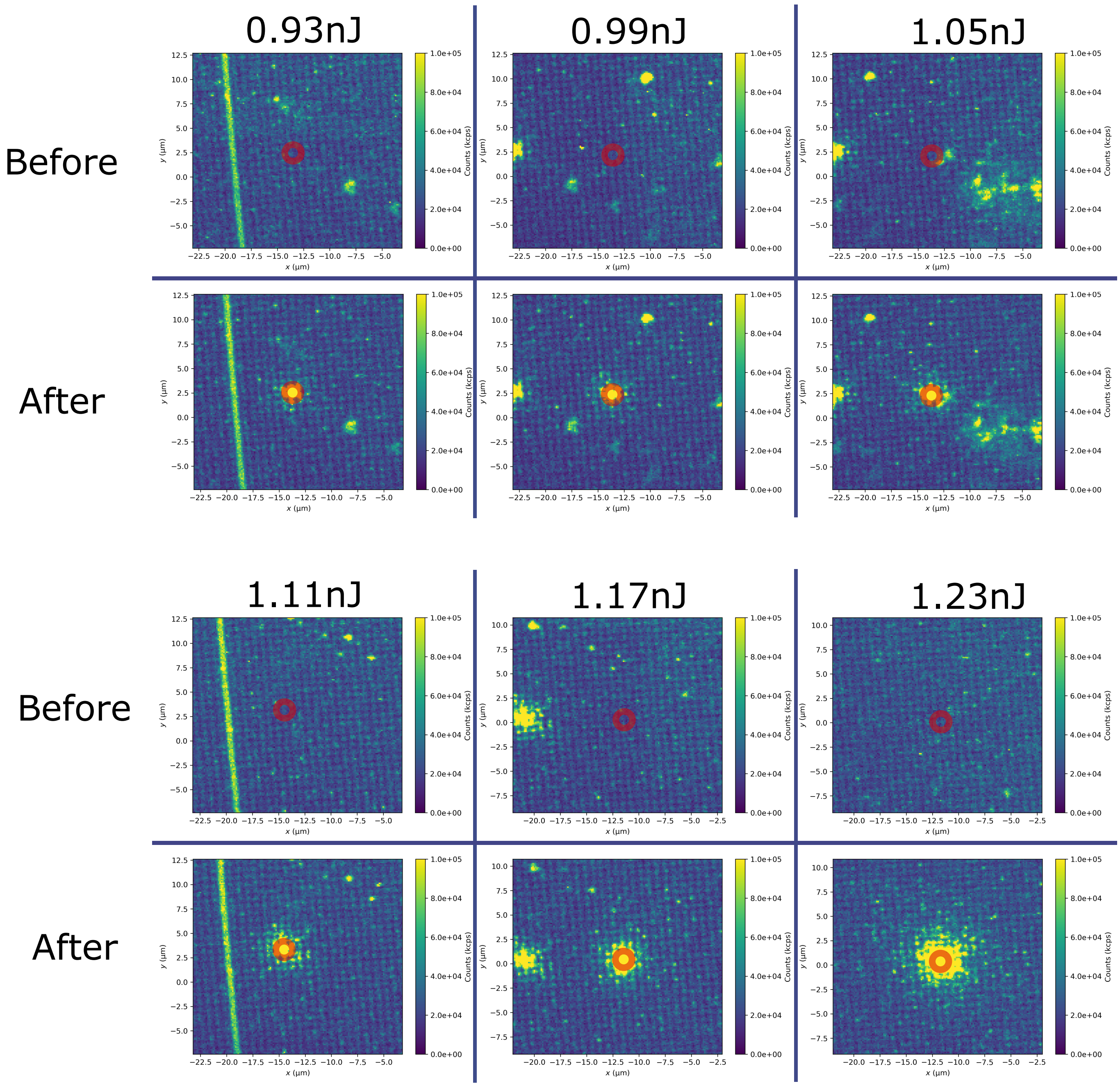

Supplementary Fig. 5: **Study of the effects of annealing laser's pulse energy**. Sets of 2D photoluminescence (PL) images are presented for a region implanted with 1000 ions per site, captured before and after 1 min of laser annealing. The images progress from left to right and top to bottom, corresponding to increasing pulse energy. The laser was focused on the red circled points in the PL maps, with a slight defocus of 1 µm from the surface.

The effects of pulse energy were also investigated, with the annealing time held constant while varying the pulse energy of the annealing laser. This study was conducted in the region implanted with 1000 ions per site, where the laser was focused on the red circled points and annealed for 1 minute. Pulse energies ranging from 0.93 nJ to 1.23 nJ were applied. The PL maps were processed as described previously, and the variations in the spread and intensity of the activated region are presented in supplementary Fig. 6.

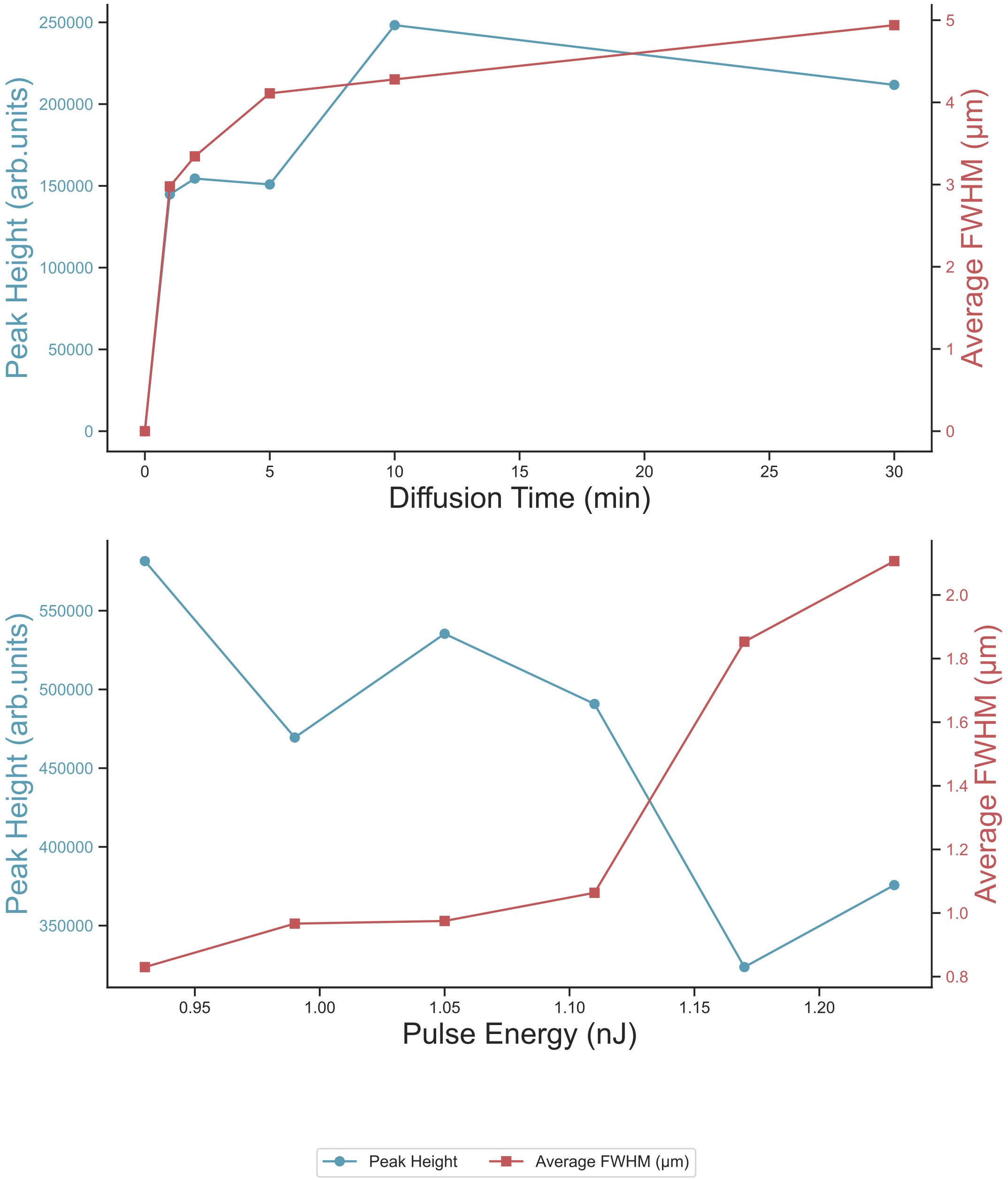

Supplementary Fig. 6: **Average full width at half maximum (FWHM) and Peak Height vs Pulse Energy and Diffusion Time**. The top graph shows the relationship between diffusion time and both the average FWHM (red) of the activated area and the peak height of the PL intensities (blue) for a region implanted with 1000 ions per site. The bottom graph displays the variation of the same parameters with respect to pulse energy.

Both the peak height and spread of the activated region increase with annealing time up to 10 minutes, after which they plateau. With a laser spot size of approximately 0.4 μm, the activated region can extend up to 5 μm, which is comparable to the exciton diffusion length in high-purity diamond at 298 K [6]. This observation supports our hypothesis that the extended laser annealing process is exciton-mediated, where femtosecond laser pulses generate hot carriers that relax to form excitons [7]. These excitons subsequently deliver energy to the lattice, facilitating the annealing process.

Increasing the pulse energy of the annealing laser has a minimal effect on the peak height, which is primarily constrained by the ion implantation dosage. However, higher pulse energy results in a slight increase in the spread of the activated region. Notably, at a pulse energy of 1.23 nJ, there is a significant drop in the fitted peak height and a marked increase in the fitted geometric mean. This behaviour is attributed to graphitisation of the diamond [8],

where the diamond lattice locally melts and recrystallises, reducing the number of vacancies and interstitials, leading to the observed changes in the fitted data.

Supplementary Note 4: Type II Sn

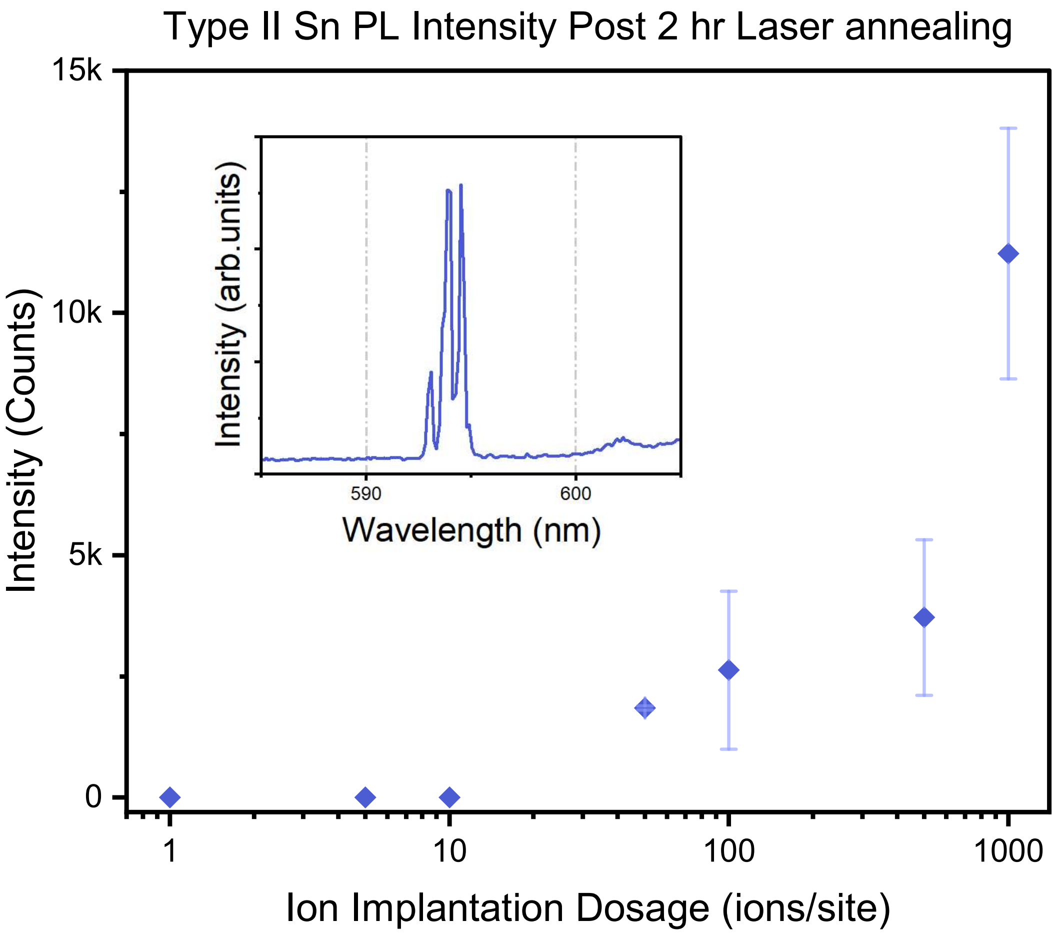

Supplementary Fig. 7: **Photoluminescence (PL) emission intensities of Type II Sn defects as a function of ion implantation dosage**. PL emission intensities (blue squares) from post 2 h laser annealing Type II Sn were obtained by integrating the spectral window from 590 to 600 nm. A representative spectrum is shown in the inset. Error bars indicate one standard error.

Supplementary Fig. 7 illustrates the relationship between implantation dosage and the PL intensities of Type II Sn defects, supplementing Fig. 1. Higher implantation dosages result in increased PL emission from Type II Sn, as more Sn ions are implanted into the diamond lattice.

To obtain details of a Type II Sn ZPL, a high resolution spectrograph grating was employed. The emission is characterised by several sharp peaks within the wavelength range of approximately 593 nm to 596 nm, with the most prominent peak centred around 594 nm. The narrow linewidths of these peaks suggest well-defined electronic transitions. The line shape and intensities of each component peak within this emission remain stable, even with sub-second acquisition windows.

Some of the PL spectra for single 'Type II Sn' centres were observed to have 4 clear peaks in their PL spectra at 5 K, with two dominant peaks and two much smaller peaks with a higher energy as shown in the top panel of supplementary Fig. 8. These findings are consistent with the data reported in the supplementary information of

Iwasaki et al.'s work [9]. The observed peaks align with a four-level energy structure, analogous to that of the SnV^- centre. The lower intensity of the higher-energy peaks can be attributed to the reduced thermal population of the upper level of a split excited state. As the temperature is increased to 20 K, these peaks grow progressively larger, following a Boltzmann distribution. The ground and excited state splittings observed are typically smaller than those of the SnV^- centre, on the order of 800 GHz and 400 GHz, respectively. However, significant variation between sites is observed, likely due to local strain effects. It should be noted that this four-peak pattern is not universally observed. Many emitters exhibit further peak splittings into doublets or more complex spectra, as seen in supplementary Fig. 9. This behaviour could be attributed to the presence of multiple emitters or local fields inducing additional small perturbations to the Hamiltonian of the system.

To calculate the polarisation of individual peaks in the ZPL, integration was performed over a window containing just one peak. When a simple four level spectrum is seen, the two large peaks are consistently polarised orthogonally to each other (see lower panel of supplementary Fig. 8), as observed with the SnV^- centre. A few-degree variation in the dipole orientations relative to the collection polariser was observed across multiple sites, as well as emitters where the linear polarisations of C and D were flipped relative to the collection polariser.

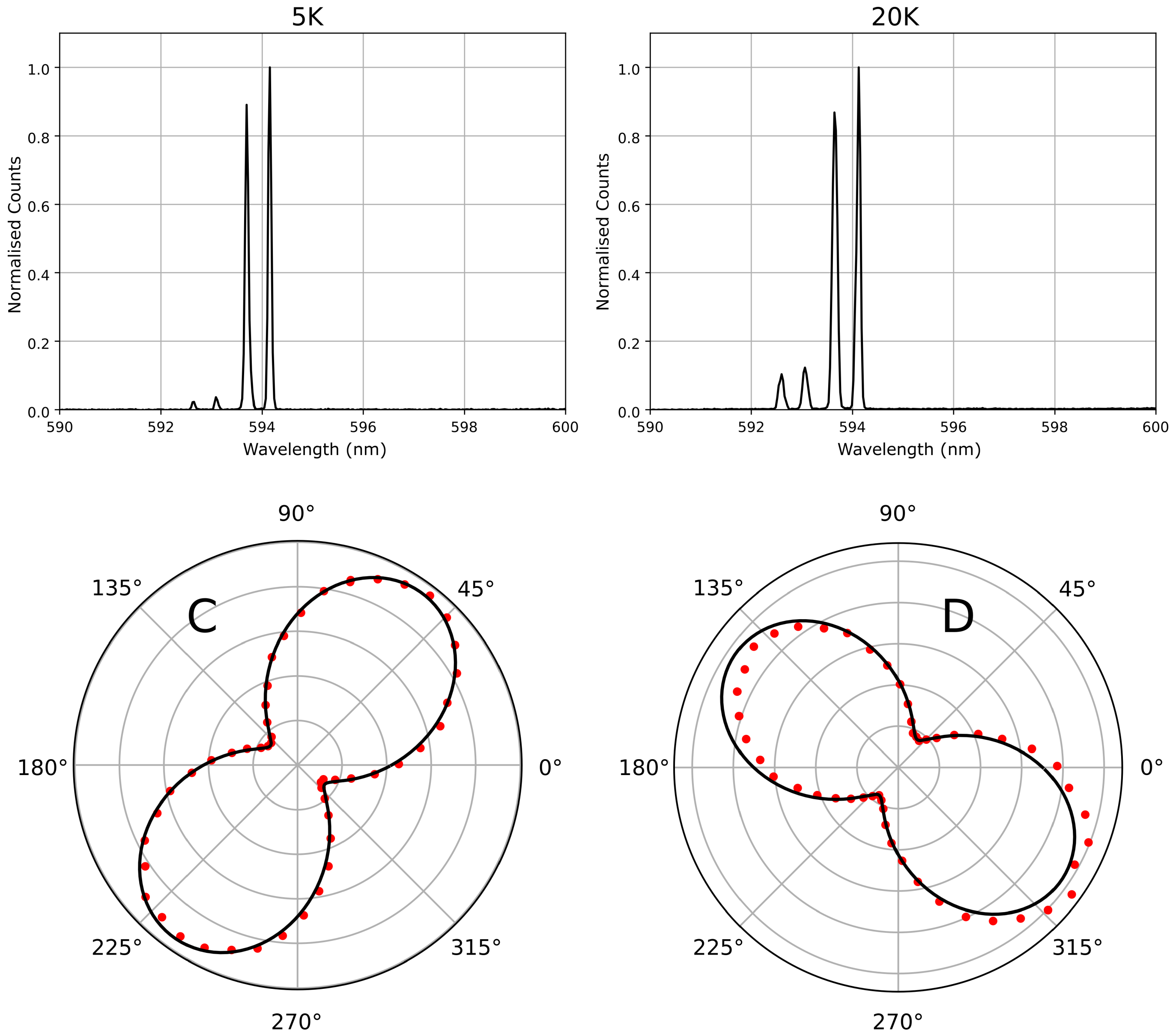

Supplementary Fig. 8: **Spectral characterisation of a single Type II Sn defect.** (Top) Spectra acquired at 5 K and 20 K using a 1200 lines/mm grating, revealing the four-level electronic structure. The higher energy peaks exhibit increased intensity with rising temperature. (Bottom) Polarisation dependence of the two primary emission peaks at 5 K, demonstrating their orthogonal nature. The data are fitted with a $\cos^2\theta$ model to determine the angle and contrast ratio..

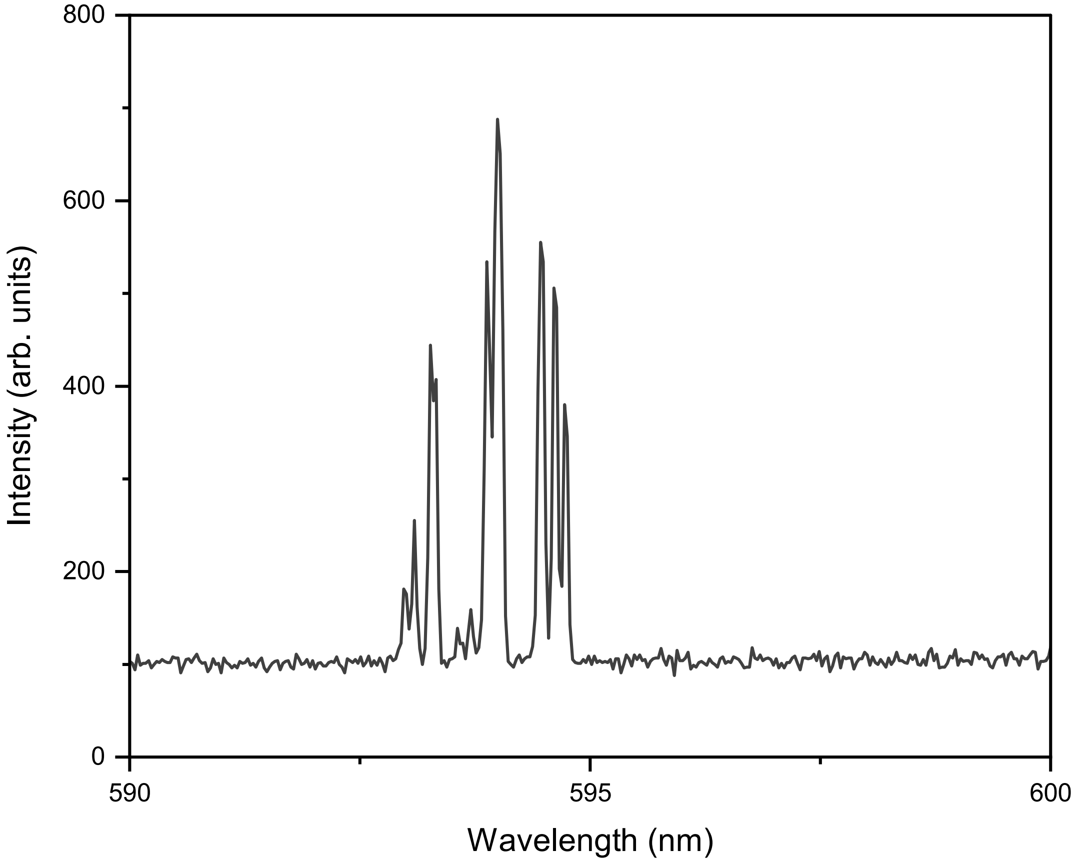

Supplementary Fig. 9: **Spectral data of a Type II Sn defect with more complicated spectral features in the zero-phonon line (ZPL), recorded using a high-resolution spectrograph grating**.

Supplementary Fig. 10 presents the results of a DFT simulation, where C_i is oriented differently compared to the configuration discussed in the main text. The simulation explores various configurations of the C_i relative to the implanted SnV defect in the diamond lattice.

The DFT calculations reveal a stable configuration where the C_i resides at a second-nearest neighbour site to the SnV^- defect, without recombining with the vacancy in the SnV^- centre. This stable state is indicated by a local energy minimum in the second configuration, showing that the system can maintain this structure without spontaneous recombination. The energy differences are presented for the migration of the C_i away from the SnV^- centre, resulting in a pure SnV^- emission.

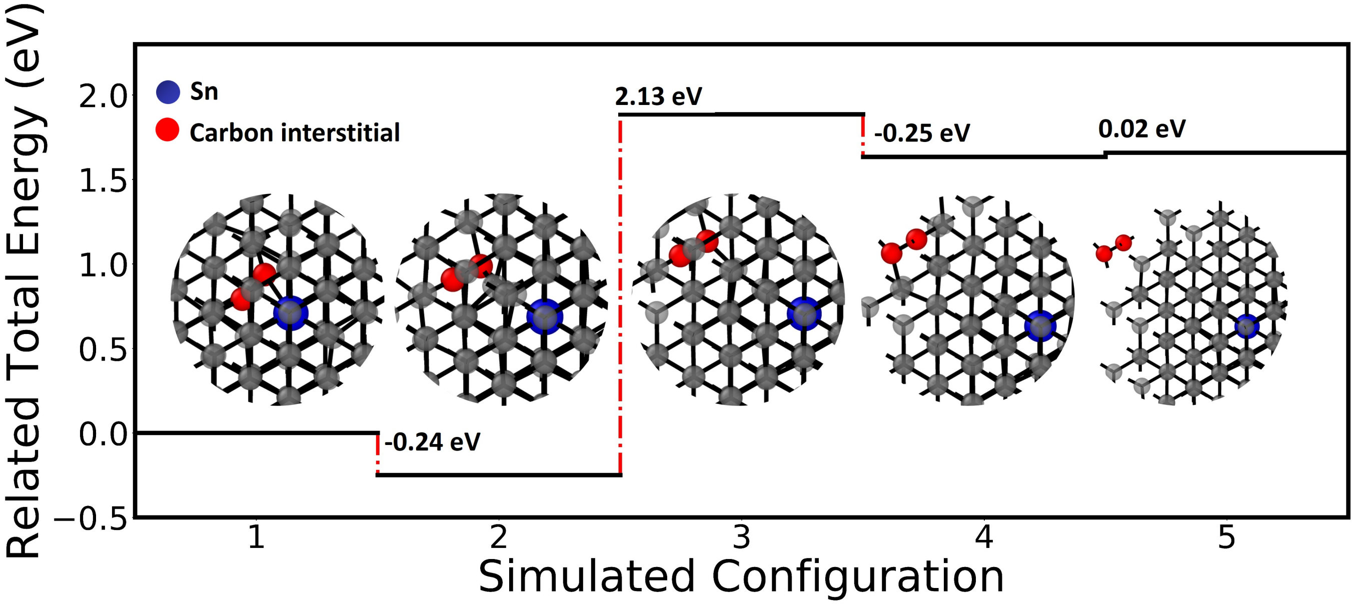

Supplementary Fig. 10: **DFT simulation of C_i configurations relative to the implanted SnV defect in diamond.** The figure illustrates the total energy of the system for various simulated configurations of the C_i. In configuration 2, the C_i is in a stable state, occupying a second-nearest neighbour site to the SnV defect without recombining with the vacancy. The energy landscape shows an energy barrier that must be overcome to move the C_i away from the SnV complex, leading to the formation of a pure SnV^- defect.

Supplementary Note 5: In-situ Monitoring

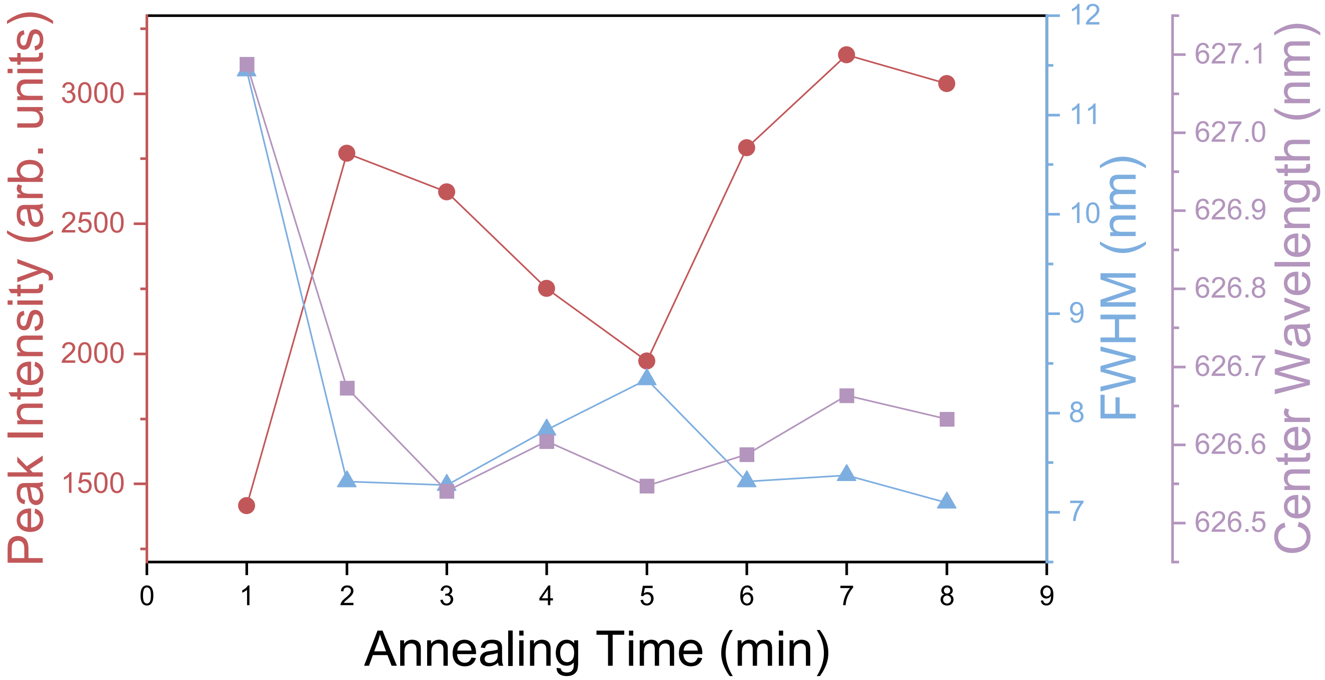

Supplementary Fig. 11: **Spectrum tracking of the SnV^- centre**. The ZPL of the SnV^- centre, as shown in Fig. 4c of the main text, is monitored over time during laser annealing. A Lorentzian fit is applied to the ZPL, with the fitting parameters extracted and plotted as a function of annealing time.

As discussed in the main text, spectral shifts, structural changes, and intensity variations were observed during the laser annealing of the SnV^- centre. Here, a Lorentzian function was fitted to the ZPL of the SnV^- centre, and the fitting parameters were extracted. The peak intensities, determined by integrating the fitted Lorentzian peaks, vary with different annealing times, suggesting that the movement of C_is around the SnV^- centre affects the degree of PL emission quenching. A correlation between peak intensity and full width at half maximum (FWHM) was observed: as the ZPL broadens, the SnV^- emission intensity decreases, indicating that the presence of a C_i suppresses the SnV^- emission. Notably, aside from the significant changes observed between 1 and 2 minutes—where both peak intensity and FWHM vary the most—the ZPL's central wavelength remains relatively stable until deactivation occurs.

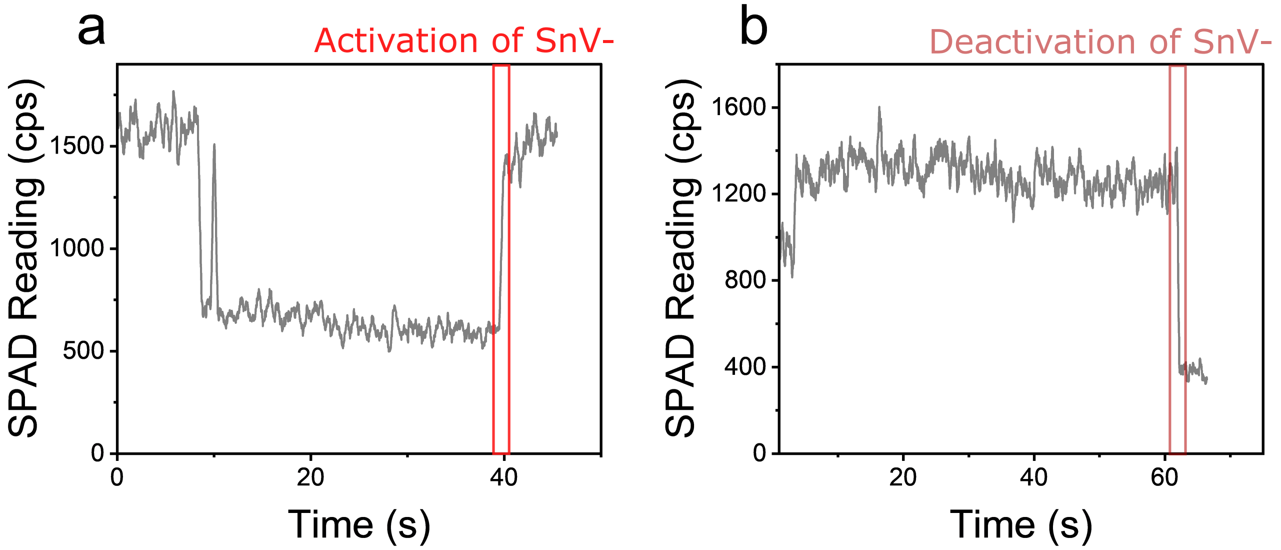

Supplementary Fig. 12: **SPAD monitoring during laser annealing**. **a**, A SPAD trace capturing the activation of a SnV^- centre during laser annealing. **b**, The corresponding trace for the deactivation of a SnV^- centre.

In addition to spectral monitoring, in-situ tracking of the laser annealing process can be performed using fluorescence feedback loop via SPAD (Single-Photon Avalanche Diode) traces. SPAD readings with a binning time of 20 ms were recorded for emitters under fs-laser annealing, while simultaneously being excited by a 532 nm continuous-wave (CW) laser using a home-built confocal microscope. The PL emission signal from the emitter was collected within the $615-625$ nm range, where the SnV^- emission is expected. Supplementary Fig. 12a shows the SPAD trace during the 4-5 minute interval in Fig. 4 a of the main text. After a further 40 seconds of annealing from the 4-minute mark, a sharp increase in SPAD counts within the 615-625 nm emission window indicates the activation of a potential SnV^- centre. The identity of the emitter was further confirmed to be SnV, as described in the main text. The initial decrease observed in the first 10 seconds of annealing, followed by a sharp spark, is not yet fully understood.

Supplementary Fig. 12b displays the SPAD trace during the 3-4 minute interval in Fig. 4 b of the main text. After an additional 60 seconds of annealing from the 3-minute mark, the SnV^- emission deactivated, evidenced by a sharp drop in SPAD counts within the $615-625$ nm emission window.

Supplementary Note 6: Laser activation of negatively charged silicon vacancy centres

This femtosecond laser activation technique can also be applied to other group-IV centres. In this study, we performed laser annealing on an ion-implanted array of 256 Si ions per site, following Poissonian statistics, with a separation of 10 µm between sites. The sample was equipped with alignment marks in the x and y directions, corresponding to the implantation sites. This allowed precise laser focusing and site-by-site annealing with in-situ tracking via a fluorescence feedback loop. The activation laser was turned off when a sharp increase in the SPAD counts was observed, indicating the activation of an emitter. To identify the type of emitters formed, corresponding spectra were recorded after laser activation. Supplementary Fig. 14 shows a typical background-subtracted emission spectrum of an emitter from the activated array, with a sharp ZPL at 737 nm, characteristic of SiV^-[10], confirming the successful activation of SiV^- centres using femtosecond laser pulses.

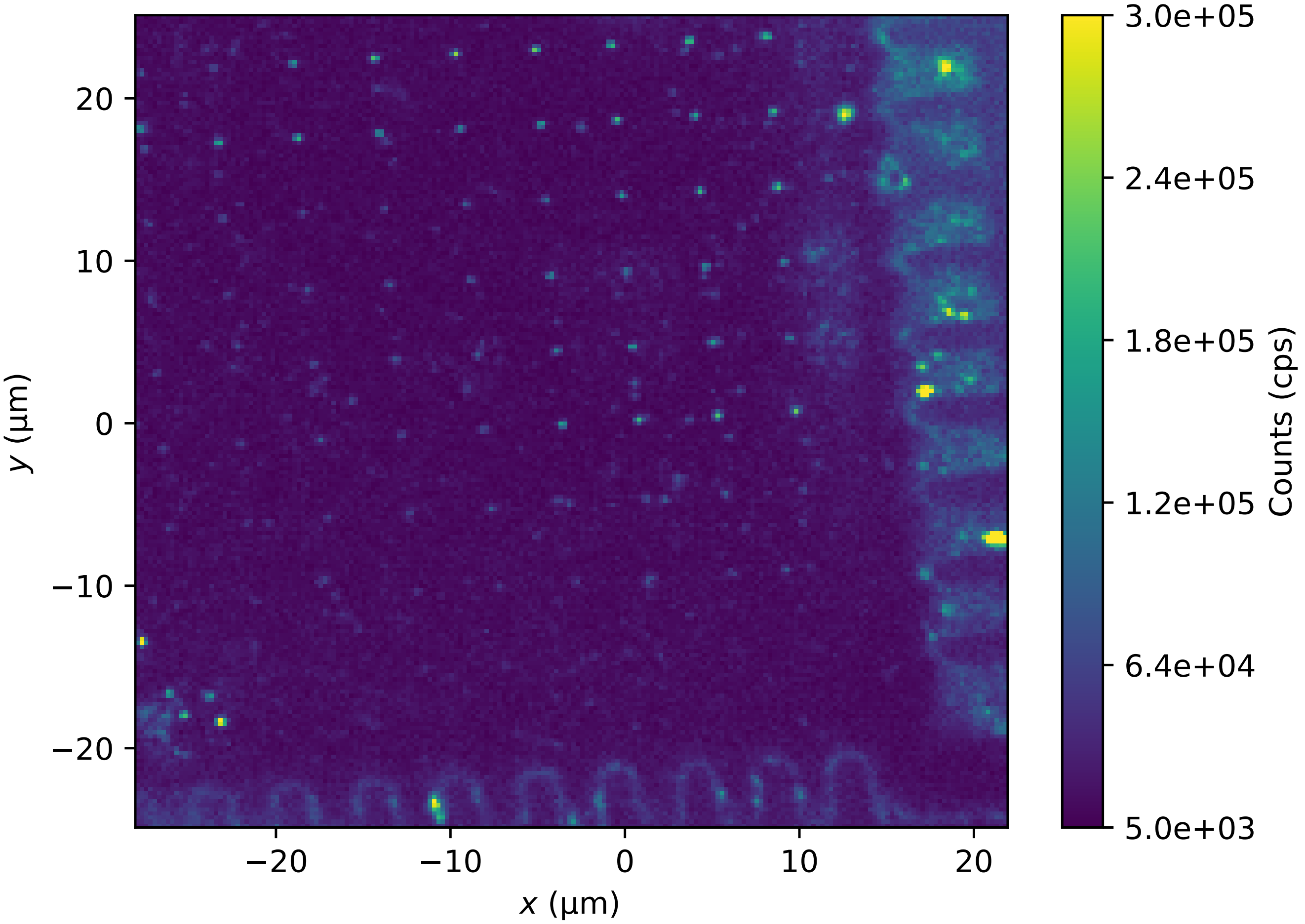

Supplementary Fig. 13: **PL image of a post-laser activation Si-implanted array**. 2D PL maps of a ion implanted with Poissonian statistics 256 Si ions in each site, laser activation performed site by site with different annealing time and pulse energy.

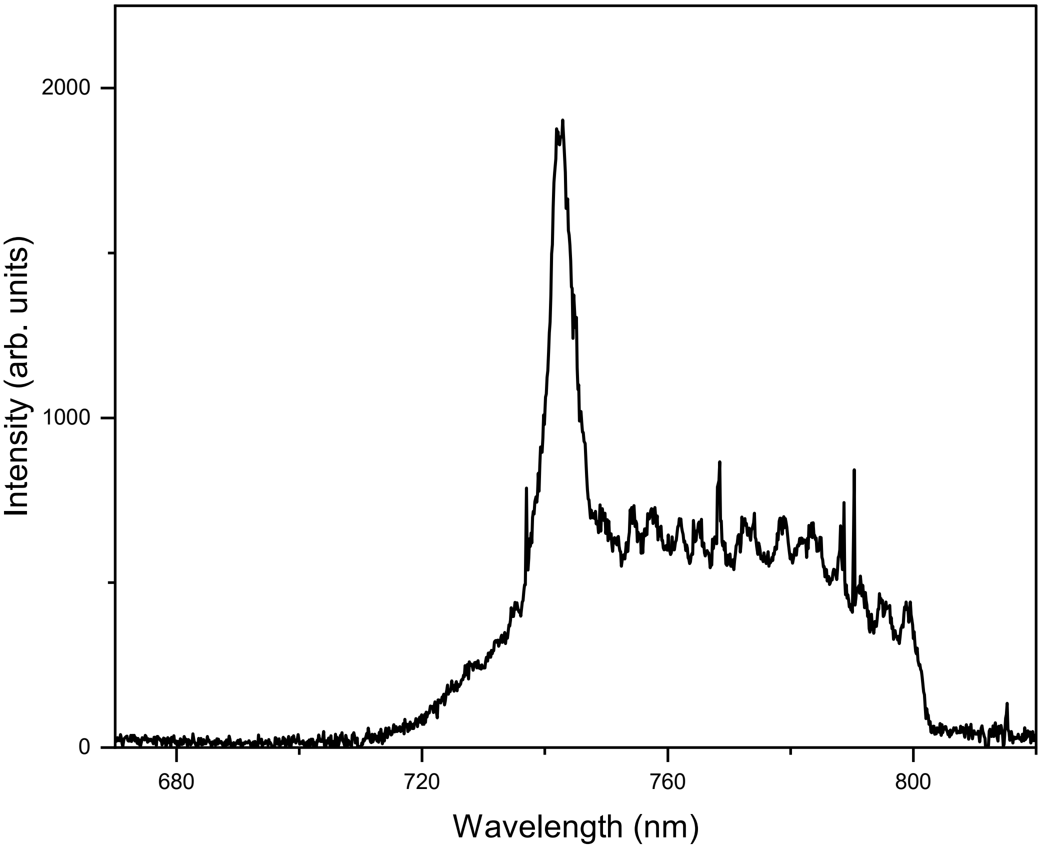

Supplementary Fig. 14: **Emission spectrum of the SiV centre.** The spectrum shows a prominent ZPL at 737 nm, confirming the activation of the SiV centre. The oscillations observed in the peaks at higher wavelengths are due to etaloning effects.

I. REFERENCES

[1] F. N. Li, P. C. Zhang, P. F. Zhang, and H. X. Wang, Thermal annealing induced graphite/diamond structure processed by high-voltage hydroxide ion treatments, Applied Surface Science **657**, 159753 (2024).

[2] J. R. Olson, R. O. Pohl, J. W. Vandersande, A. Zoltan, T. R. Anthony, and W. F. Banholzer, Thermal conductivity of diamond between 170 and 1200 k and the isotope effect, Phys. Rev. B **47**, 14850 (1993).

[3] R. R. Reeber and K. Wang, Thermal expansion, molar volume and specific heat of diamond from 0 to 3000k, Journal of Electronic Materials **25**, 63 (1996).

[4] V. V. Kononenko, E. V. Zavedeev, M. I. Latushko, and V. I. Konov, Observation of fs laser-induced heat dissipation in diamond bulk, Laser Physics Letters **10**, 036003 (2013).

[5] M. Kozák, F. Trojánek, B. Dzurňák, and P. Malý, Two- and three-photon absorption in chemical vapor deposition diamond, JOSA B **29**, 1141 (2012).

[6] H. Morimoto, Y. Hazama, K. Tanaka, and N. Naka, Exciton lifetime and diffusion length in high-purity chemical-vapor-deposition diamond, Diamond and Related Materials **63**, 47 (2016), 9th International Conference on New Diamond and Nano Carbons – NDNC 2015.

[7] B. Griffiths, A. Kirkpatrick, S. S. Nicley, R. L. Patel, J. M. Zajac, G. W. Morley, M. J. Booth, P. S. Salter, and J. M. Smith, Microscopic processes during ultrafast laser generation of frenkel defects in diamond, Phys. Rev. B **104**, 174303 (2021).

[8] Y.-C. Chen, P. S. Salter, S. Knauer, L. Weng, A. C. Frangeskou, C. J. Stephen, S. N. Ishmael, P. R. Dolan, S. Johnson, B. L. Green, G. W. Morley, M. E. Newton, J. G. Rarity, M. J. Booth, and J. M. Smith, Laser writing of coherent colour centres in diamond, Nature Photonics **11**, 77 (2017).

[9] T. Iwasaki, Y. Miyamoto, T. Taniguchi, P. Siyushev, M. H. Metsch, F. Jelezko, and M. Hatano, Tin-vacancy quantum emitters in diamond, Phys. Rev. Lett. **119**, 253601 (2017).

[10] T. Müller, C. Hepp, B. Pingault, E. Neu, S. Gsell, M. Schreck, H. Sternschulte, D. Steinmüller-Nethl, C. Becher, and M. Atatüre, Optical signatures of silicon-vacancy spins in diamond, Nature Communications **5**, 3328 (2014).
